# Supplementary material for: The interplay between body mass index, motivation for food consumption, and noncommunicable diseases in the European population: A cross-sectional study
Source: PLoS One. 2025 May 14;20(5):e0322454. doi: 10.1371/journal.pone.0322454 (PMC12077776; doi:10.1371/journal.pone.0322454)
Supplement: S1 Table — (DOCX) [file pone.0322454.s001.docx]

Inclusivity in global research

PLOS’ policy on inclusivity in global research aims to improve transparency in the reporting of research performed outside of researchers’ own country or community and ensures that PLOS publications reporting global research adhere to high standards for research ethics and authorship. Authors of relevant research articles may be asked to complete the questionnaire below, which outlines ethical, cultural, and scientific considerations specific to inclusivity in global research. This questionnaire may be requested when researchers have travelled to a different country to conduct research, if research uses samples collected in another country, research with Indigenous populations or their lands, or if research is on cultural artefacts. Researchers travelling to another country solely to use laboratory equipment will not normally be required to complete the questionnaire. However, the questionnaire can be requested at the journal’s discretion for any submission – if you have been requested to complete this questionnaire by the PLOS journal you submitted to, please do so.

Please complete the questionnaire below and include this as a Supporting Information file with your manuscript. Note that if your paper is accepted for publication, this checklist will be published with your article in the supporting information files. Please ensure that you reference the checklist in the main body of your manuscript. We suggest adding a subsection ‘Inclusivity in global research’ to your Methods section and adding the following sentence: “Additional information regarding the ethical, cultural, and scientific considerations specific to inclusivity in global research is included in the Supporting Information (SX Checklist)”

The questions have been designed to be applicable to a wide range of study types, and there are subsections for both human subjects research and non-human subjects research. If any of the questions are not relevant to your research please mark them as “N/A” as appropriate.

**Ethical considerations, permits and authorship**

*This section is applicable to all research types.*

Provide details as to who granted permissions and/or consent for the study to take place in the Methods section of your manuscript. This should include the names of **all** ethics boards, governmental organizations, community leaders or other bodies that provided approval for the study. If individuals provided approval refer to these people by their role or title but do not list their name(s).

Reported on page number: 9.

Ethical approval was granted by the Ethical Committee of the Polytechnic Institute of Viseu (registration number 04/2017), the project holder. The study adhered to the ethical guidelines of the Declaration of Helsinki, and was approved for ethics purposes and for application in the following institutions of each participating country, in addition to that of the project holder: General Hospital Zadar, Croatia; Alexander Technological Educational Institute of Thessaloniki (ATEITh), School of Agriculture Technology, Food Technology and Nutrition, Greece; Hungarian Chamber of Agriculture, Hungary; University of Parma, Italy; Latvia University of Agriculture, Faculty of Food Technology, Department of Food Technology, Latvia; The Netherlands Food Quality and Design Group, Wageningen University & Research, The Netherlands; Wroclaw University of Environmental and Life Sciences, Faculty of Food Science, Poland; University of Medicine and Pharmacy Tirgu-Mures, Faculty of Medicine, Romania; University of Belgrade, Faculty of Agriculture, Republic of Serbia; University of Primorska, Faculty of Health Sciences, Department of Nutritional Counselling-Dietetics, Slovenia. Approvals from all Ethics Committees were obtained before data collection began.

If there were any deviations from the study protocol after approval was obtained please provide details of  these changes in Methods section of your manuscript.

No

Did this study involve local collaborators that are residents of the country where the research was conducted or members of the community studied? If you do not have any authors from said communities, please provide an explanation for this below.

We have included at least one author from each local team to ensure that our authorship is representative and comprehensive. This study is part of a larger European collaborative project involving several countries and regions. Each participating country contributed through local experts and community representatives familiar with regional conditions and needs. This approach was deliberately chosen to increase the research's cultural, contextual, and scientific relevance.

By involving local collaborators, we ensured that the study reflected different perspectives and was better aligned with the communities studied. In addition, local teams were actively involved in all stages of the research process, including study design, data collection, analysis, and interpretation of results. This collaborative model emphasizes the inclusivity and representativeness of our work.

Everyone listed as an author should meet PLOS’ criteria for authorship and all individuals who meet these criteria should be included in the author byline, rather than the acknowledgements. For further information please see the journal’s Authorship Policy.

**Human subjects research (e.g. health research, medical research, cross-cultural psychology)**

Did you obtain written informed consent from a representative of the local community or region before the research took place? How did you establish who speaks for the community? Details of written informed

consent obtained from study participants should be reported separately in the Methods section of your manuscript.

Yes, informed consent was obtained from representatives of the local communities or regions where the research was conducted. Approval was obtained for the entire study and separately in each country. Representatives were selected based on their recognized leadership roles in their communities or their connections to relevant local organizations and institutions. This approach was essential for the ethical and culturally appropriate conduct of our research.

Participants who agreed to participate in the study provided verbal informed consent. For each participant agreeing to participate in the study, the researcher who conducted the informed consent procedure filled out the oral consent template and signed the document in the presence of the participant to confirm his consent.

Details of the procedures for obtaining voluntary participation, can be found in the section Materials and Methods on the pages 9-10.

How did members of the local community provide input on the aims of the research investigation, its methodology, and its anticipated outcome(s)?

Local community members were actively involved in shaping the objectives, methodology and expected outcomes of the research investigation. Their input was sought through a series of consultations and participatory workshops during the planning phase of the study and project. As part of these consultations, discussions were held with community representatives, local organizations and stakeholders to identify key issues and priorities relevant to the research. Feedback from these meetings was integral to defining the research questions, refining the study design and ensuring that the methodology was culturally sensitive and contextually appropriate.

When engaging with the local community, how did you ensure that the informed consent documents and other materials could be understood by local stakeholders?

Before recruitment, participants were informed of the study's goals and assured that participation was voluntary, and their responses would remain anonymous and confidential. All the information regarding research was given in easily understandable language. After being provided with all the information needed to make an informed decision, participants were given a reasonable amount of time to consider participation in the study. During that period, the researchers were available for any questions related to the study.

Will the findings of the research be made available in an understandable format to stakeholders in the community where the study was conducted (e.g. via a presentation, summary report, copies of publications, etc.)? Please provide details of how this will be achieved.

Yes, the research findings will be shared with stakeholders in the community where the study was conducted in an accessible and understandable format.

We plan to organize presentations in the community where key findings will be presented in a clear and concise manner using visual aids and examples relevant to the local context. In addition, summaries in the local language(s) will be distributed to community representatives, local organizations and other stakeholders.

For further dissemination, copies of relevant publications will be made available to local stakeholders, together with simplified summaries tailored to a non-academic audience. To ensure inclusiveness, digital versions of the summaries will be disseminated via community networks, emails and common news platforms in the region.

**Non-human subjects research using specimens/ animals collected as part of the study, or those housed in archival collections. Examples include archaeology, paleontology, botany and zoology.**

Did the permission you obtained from a local authority to perform the study include an agreement on access to outputs and benefit sharing? This may include procedures to enable fair distribution of the benefits and resources arising from the research performed. Please include any details of Prior Informed Consent and Benefit Sharing Agreements obtained. These may be required by field-specific regulations, for example the Convention on Biological Diversity (CBD) and the associated Nagoya Protocol.

N/A

If the material used in your study was imported, please A) provide the year it was imported and B) indicate whether permits were obtained to import/export the materials used, C) provide details of any permits obtained. If this information is not available, please indicate this.

N/A

If you used archival specimens, please state how the material used in your study was acquired by the institute it is held in and provide details of any permits obtained for the original excavations/ sample collection. If this information is not available, please indicate this.

N/A

How was the potential cultural significance of the materials collected in your study to local communities considered in your research design? Were Indigenous peoples and/or local researchers and institutions involved with archaeological excavations / collection of specimens? If so, please provide a description of their involvement.

N/A

If your manuscript includes photographs of human remains please indicate whether authors obtained permission from descendants or affiliated cultural communities to do so.

N/A.
