# Supplementary material for: The interplay between body mass index, motivation for food consumption, and noncommunicable diseases in the European population: A cross-sectional study
Source: PLoS One. 2025 May 14;20(5):e0322454. doi: 10.1371/journal.pone.0322454 (PMC12077776; doi:10.1371/journal.pone.0322454)
Supplement: S2 Table — (DOCX) [file pone.0322454.s002.docx]

| S2 Table Sociodemographic characteristics of European population (N=9,036). | | | | | | | | | | | | | | |
| --- | --- | --- | --- | --- | --- | --- | --- | --- | --- | --- | --- | --- | --- | --- |
|  | | Croatia  (N=1,538) | Greece  (N=498) | Hungary  (N=500) | Italy  (N=541) | Latvia  (N=634) | Lithuania  (N=507) | Netherlands  (N=521) | Poland  (N=583) | Portugal  (N=1304) | Romania  (N=821) | Serbia  (N=498) | Slovenia  (N=1,091) | Overall p |
| Age (years), Mdn (IQR) | | 32.0  (25.0) | 23.0 (22.0) | 43.0 (18.0) | 40.0 (19.0) | 36.0 (19.0) | 25.0  (24.0) | 29.0  (25.0) | 30.0 (18.0) | 37.0 (26.0) | 38.0 (24.0) | 23.0  (7.0) | 30.0 (17.0) | <0.001† |
| Gender,  N (%) | Male | 486  (31.6) | 181  (36.3) | 247  (49.4) | 187  (34.6) | 118  (18.6) | 125  (24.7) | 70  (13.4) | 139 (23.8) | 431  (33.1) | 255  (31.1) | 175  (35.1) | 137  (12.6) | <0.001* |
|  | Female | 1052  (68.4) | 317  (63.7) | 253  (50.6) | 354  (65.4) | 516  (81.4) | 382  (75.3) | 451  (86.6) | 444  (76.2) | 873  (66.9) | 566  (68.9) | 323  (64.9) | 954  (87.4) |  |
| Environment,  N (%) | Urban | 1318  (85.7) | 474  (95.2) | 358  (71.6) | 481  (88.9) | 542  (85.5) | 457  (90.1) | 497  (95.4) | 514 (88.2) | 1092 (83.7) | 707  (86.1) | 466  (93.6) | 747  (68.5) | <0.001* |
|  | Rural | 220  (14.3) | 24  (4.8) | 142  (28.4) | 60  (11.1) | 92  (14.5) | 50  (9.9) | 24  (4.6) | 69  (11.8) | 212  (16.3) | 114  (13.9) | 32  (6.4) | 344  (31.5) |  |
| Marital status,  N (%) | In marriage | 724  (47.1) | 154  (30.9) | 324  (64.8) | 357  (66.0) | 360  (56.8) | 242  (47.7) | 257  (49.3) | 385 (66.0) | 654  (49.8) | 524  (63.8) | 108  (21.7) | 688  (63.1) | <0.001* |
|  | Single/divorced/widowed | 814  (52.9) | 344  (69.1) | 176  (35.2) | 184  (34.0) | 274  (43.2) | 265  (52.3) | 264  (50.7) | 198 (34.0) | 650  (50.2) | 297  (36.2) | 390  (78.3) | 403  (36.9) |  |
| Education level,  N (%) | Primary and high school | 716  (46.6) | 67  (13.5) | 339  (67.8) | 271  (50.1) | 145  (22.9) | 229  (45.2) | 113  (21.7) | 109 (18.7) | 561  (43.0) | 182  (22.2) | 151  (30.3) | 534  (48.9) | <0.001* |
|  | University | 822  (53.4) | 431  (86.5) | 161  (32.2) | 270  (49.9) | 489  (77.1) | 278  (54.8) | 408  (78.3) | 474 (81.3) | 743  (57.0) | 639  (77.8) | 347  (69.7) | 557  (51.1) |  |
| Working status,  N (%) | Employed | 998  (64.9) | 214  (43) | 388  (77.6) | 425  (78.6) | 536  (84.5) | 248  (48.9) | 287  (55.1) | 438  (75.1) | 859  (65.9) | 591  (72) | 147  (29.5) | 778  (71.3) | <0.001* |
|  | Unemployed/ retired/student | 540  (35.1) | 284  (57) | 112  (22.4) | 116  (21.4) | 98  (15.5) | 259  (51.1) | 234  (44.9) | 145  (24.9) | 445  (34.1) | 230  (28) | 351  (70.5) | 313  (28.7) |  |
| Note: Mdn (IQR)= Median (Interquartile Range); N (%)=absolute number (percentage); † Kruskal Wallis Test ; *Chi Square test. | | | | | | | | | | | | | | |
