# Supplementary material for: The interplay between body mass index, motivation for food consumption, and noncommunicable diseases in the European population: A cross-sectional study
Source: PLoS One. 2025 May 14;20(5):e0322454. doi: 10.1371/journal.pone.0322454 (PMC12077776; doi:10.1371/journal.pone.0322454)
Supplement: Attachment — Submitted filename: Response to Reviewers.docx [file pone.0322454.s005.docx]

**Reviewer's Responses to Questions**

**1. Is the manuscript technically sound, and do the data support the conclusions?**

The manuscript must describe a technically sound piece of scientific research with data that supports the conclusions. Experiments must have been conducted rigorously, with appropriate controls, replication, and sample sizes. The conclusions must be drawn appropriately based on the data presented.

**Reviewer #1: Yes**

**Authors:** Thank you for your response.

**2. Has the statistical analysis been performed appropriately and rigorously?**

**Reviewer #1: Yes**

**Authors:** Thank you for your response.

**3. Have the authors made all data underlying the findings in their manuscript fully available?**

The PLOS Data policy requires authors to make all data underlying the findings described in their manuscript fully available without restriction, with rare exception (please refer to the Data Availability Statement in the manuscript PDF file). The data should be provided as part of the manuscript or its supporting information, or deposited to a public repository. For example, in addition to summary statistics, the data points behind means, medians and variance measures should be available. If there are restrictions on publicly sharing data—e.g. participant privacy or use of data from a third party—those must be specified.

**Reviewer #1: Yes**

**Authors:** Thank you for your response.

**4. Is the manuscript presented in an intelligible fashion and written in standard English?**

PLOS ONE does not copyedit accepted manuscripts, so the language in submitted articles must be clear, correct, and unambiguous. Any typographical or grammatical errors should be corrected at revision, so please note any specific errors here.

Reviewer #1: Yes

**Authors:** Thank you for your response.

**5. Review Comments to the Author**

Please use the space provided to explain your answers to the questions above. You may also include additional comments for the author, including concerns about dual publication, research ethics, or publication ethics. (Please upload your review as an attachment if it exceeds 20,000 characters)

**Reviewer #1: PONE-D-24-44681**

This paper describes a large cross sectional cohort study of European adults, examining associations between self-reported emotional state, food intake, motivation and various non-communicable disease related outcomes. It is an excellent paper overall.

**Response:** Thank you for your kind and encouraging feedback. We sincerely appreciate your recognition of our work and are delighted that you found the paper to be of high quality. Your insights and support mean a great deal to us.

I would have the following comments.

1. Firstly, in the introduction and throughout, it is clear the authors have a “behaviourist” view on obesity and why it affects some people more than others. For example, they state that “… maintaining health requires strong motivation combined with positive lifestyle habits such as eating healthy foods…”. I think that this could be disputed, because it infers that people with obesity lack motivation, or indeed that thin people must be highly motivated. I’m sure that this is not what the authors intend, but it would really strengthen the relevance and acceptability of the papers very interesting findings if the authors acknowledged more the very profound biological and genetic basis for variations in the complex neurobehavioural responses to what has become an increasingly obesogenic environment. Within that, the nuances of emotional state are critically important considerations, but I’d love to see more consideration of this issue throughout.

**Response Ad 1.** Thank you for your valuable comments. We deeply appreciate your thoughtful feedback and have carefully followed your guidance in revising the introduction and throughout the manuscript. Your suggestions have been thoughtfully considered to enhance the quality and clarity of our work.

Among other things, after the sentence“*… maintaining health requires strong motivation combined with positive lifestyle patterns such as eating healthy foods…”* we added the new sentence ***“****Hence,, it is important to recognize that the development of obesity is not only due to individual behaviors or lack of motivation, as genetic, epigenetic, neurobiological and environmental factors also play an important role in weight gain and its effects on different individuals”*

We have reworded the introduction in line with your suggestions and ensured that this approach is consistently maintained throughout the whole manuscript.

2. Furthermore, I’d be careful of the use of the term “habits”. Arguably the strong biological basis for obesity makes the various implicated behaviour patterns more than mere habits. Perhaps consider that variations in MHB and EMFC could be strongly influenced by genes, biology and pathophysiology, rather than by affected individuals just not thinking in the right way or not making enough effort. I think this is really important for this paper, in order that it has its deserved impact. Consider the famous paper in 2018 by Rogers in the Lancet, “telling us what did not cause the obesity epidemic”.

**Response Ad 2.** Thank you for this comment. We agree with you. We have replaced the term habit with a more appropriate term or corrected the sentences. We have also emphasized the importance of genetic, biological and pathophysiological mechanisms, as you recommended. We have done this throughout the manuscript. We have carefully reviewed the article by Rogers in the Lancet 2018 and other similar references and add them to the reference list.

3. It is not clear why the authors dichotomised BMI.

**Response Ad 3.** Thank you for your question regarding the dichotomization of BMI at a threshold of 25. This cutoff was chosen because it is a well-established and standardized criterion set by the World Health Organization (WHO) to distinguish between normal weight (BMI < 25) and overweight (BMI ≥ 25) in adults. Using this threshold ensures consistency with previous research and aligns with public health guidelines, making it easier to compare findings and apply them in both clinical and epidemiological settings. Additionally, dichotomizing BMI at this point helps structure the analysis of its relationship with other variables in the study. We have added this explanation to the statistical analysis section for clarity.

*“*

*“BMI was dichotomized according to the World Health Organization (WHO) classification, as described in Study design and participants section, to ensure adherence to established public health standards and to facilitate the interpretation of results. However, it is important to note that a BMI of 25.0 kg/m2 or higher encompasses both overweight and obesity, with obesity defined as a BMI of 30.0 kg/m2 or greater. Conversely, a BMI below 25.0 kg/m2 does not necessarily indicate normal body weight. For instance, a BMI of <18.5 kg/m2, reflects malnutrition rather than a healthy weight. This highlights the need to interpret BMI values within the broader context of individual health and nutritional status. NCDs variables were also dichotomized (presence or absence of disease) to ensure interpretability within the logistic regression model. Predictors in all these models included the same variables as in the linear regression models mentioned above. Referent groups were also defined as in previous regression models. Beta values were converted into odds ratios with 95% confidence intervals (lower and upper bounds).*

*To minimize statistical bias, all predictors were simultaneously included in the regression models. In certain models, the primary outcome variables were also incorporated as predictors in the analysis.”*

4. Can I check are the regression analyses in table 2 based on the treatment of BMI as a continuous rather than a categorical variable?

**Response Ad 4.** Table 2 shows a linear regression model, and BMI is included as a continuous outcome variable. We examined BMI as an outcome variable in its relationship with motivation. According to the principles of statistics, linear regression requires the dependent variable (outcome) to be continuous. This ensures the fulfillment of key model assumptions, including the linearity of relationships between variables. In linear regression BMI as outcome variable should be continuous and because of that we left it as continuous, while we dichotomized it in the logistic regression.

For clarity, we add the sentence in statistical method *“BMI was included as a continuous variable in all liner regression models.”*

5. Also, the statement that “Participants with BMI ≥ 25.0 kg/m^2^ had an increased risk of… development of obesity” seems tautological (and inconsistent with the cross-sectional nature of the study). Of course, those with a high BMI were more likely to have obesity.

**Response Ad 5.** Thank you for these comments. We agree that in the context of a cross-sectional study, the OR (Odds ratio) represents the probability of an association between an exposure and an outcome, rather than a measure of risk, as the study design does not establish causality. For clarity, we have followed your recommendation and rephrased “risk” to “likelihood” or “probability” of an association between an exposure and an outcome.

6. Rather than “Association between body mass index and incidence of chronic noncommunicable diseases”, it should be prevalence (as this is cross-sectional).

**Response Ad 6.** Thank you. We deleted the word “incidence”.

7. Again, the statement that “Participants with obesity had almost 18 times higher odds of additionally increasing their BMI” makes no sense to me.

**Response Ad 7.** We reformulated this sentence *“Participants with obesity were more likely to have an additional increase in BMI [OR=17.89 (95% CI 2.29-26.03); p<0.001] and were at higher risk of developing most NCDs.”*

8. I don’t think the between country comparisons offer much in the paper relevant to the hypothesis in hand. It is noteworthy though that 7.2% of Serbian adults have a BMI under 18.5 kg/m^2^.

**Response Ad 8.** Thank you for your thoughtful feedback. We appreciate your pointing out the limited relevance of country comparisons in the context of our hypothesis. Although these comparisons do not directly address the hypotheses, we included them to provide a broader context and explore possible national differences that might influence the relationships between BMI, lifestyle, food consumption, socioeconomic and emotional states. As you mentioned, the notable percentage of Serbian adults with a BMI below 18.5 kg/m^2^ is an interesting finding that could be explored further in the context of broader demographic trends. We have added these comparisons for Serbia. We also add the results from Portugal, where the prevalence of normal BMI is highest “*Participants from Portugal had the highest proportion of people with a normal BMI (79.9%) compared to other countries, while 7.2% of Serbian adults had a BMI below 18.5 kg/m^2^.“*

**PLOS authors have the option to publish the peer review history of their article (what does this mean?).** If published, this will include your full peer review and any attached files.

If you choose “no”, your identity will remain anonymous but your review may still be made public.

Do you want your identity to be public for this peer review? For information about this choice, including consent withdrawal, please see our Privacy Policy.

**Reviewer #1**: Yes: Francis Finucane
