# Supplementary material for: The interplay between body mass index, motivation for food consumption, and noncommunicable diseases in the European population: A cross-sectional study
Source: PLoS One. 2025 May 14;20(5):e0322454. doi: 10.1371/journal.pone.0322454 (PMC12077776; doi:10.1371/journal.pone.0322454)
Supplement: Attachment — Submitted filename: Response to Reviewers R2.docx [file pone.0322454.s006.docx]

**PONE-D-24-44681R1**

The interplay between body mass index, motivation for food consumption, and noncommunicable diseases in the European population: a cross-sectional study

**Additional Editor Comments:**

(PLACE INSERTS IN A DIFFERENT COLOR FONT TO IDENTIFY CHANGES IN THE ARTICLE)

PUT IN RED LETTERS - ANSWER THE QUESTIONS BELOW POINT BY POINT.

PLEASE INCLUDE ALL REQUESTS IN THE MANUSCRIPT.

We followed the instructions and acted accordingly. Thank you.

Include in article

**Point 0. Please correct grammatical and spelling errors**

Ad 0. Thank you for your comment. The paper has been grammatically and stylistically revised.

**Point 1. Abstract**

Conclusions: State only what your study found; do not include extraneous information not backed up by the results.

**Response Ad 1.**

Thank you for your comment. We reformulated the conclusion: *“**These results underscore the need for targeted public health interventions that address emotional eating behaviors and promote healthier coping strategies to mitigate the risk of obesity and related health complications.”*

**Point 2. Discussion**

Compare and contrast your study with others in the most relevant world literature, particularly the recent literature.

***Response Ad 2*.**

Thank you for your valuable feedback. We have expanded the discussion by explicitly comparing our findings with the most relevant recent literature. Additionally, we have enhanced other sections of the paper by incorporating the latest research, clearly linking it to our results to provide a more comprehensive analysis and strengthen the connection between our findings and the current new evidence.

***Point 3****.* What new information is sufficient to modify existing clinical practice

***Response Ad 3.***

Thank you for this comment. We have added the reformulated paragraph at the end of the Discussion: „This study supports the integration of psychological support with lifestyle interventions to enhance MHB, combat emotional eating and weight gain, and improve the clinical management of NCDs. Considering the role of emotional regulation in eating behavior and implementing tailored interventions that combine psychological support with nutritional counseling may lead to more personalized, holistic treatment approaches [29,34,35,38].”

***Point 4****.* What are the conclusions and implications for current practice, and particularly for future research that may have a significant impact on clinical decisions?

***Response Ad 4.***

Thank you for this comment. We have added the following at the end of the Discussion: *“**Incorporating these findings into clinical practice could enable healthcare providers to offer more comprehensive strategies for obesity treatment and prevention, ultimately improving long-term patient outcomes. Future research should further investigate how emotional, sociodemographic, and psychological factors influence MHB, weight gain risk, and the development of NCDs. The results of this study contribute to shaping public health policy by providing evidence for integrated interventions that address lifestyle, psychological, and behavioral factors in the prevention and treatment of obesity and related diseases.“*

***Point 5.*** How can this study affect public policies related to health?

***Response Ad 5.***

Thank you for this comment. We have added the sentence*:* “The results of this study contribute to shaping public health policy by providing evidence for integrated interventions that address lifestyle, psychological, and behavioral factors in the prevention and treatment of obesity and related diseases.”

***Point 6.*** What does this study add to the literature?

***Response Ad 6.***

Thank you for this comment. We have added the sentence: *„This study expands the literature by providing large-scale, cross-national evidence on the relationship between BMI, EMFC, and health outcomes, demonstrating that emotional eating is a significant predictor of obesity and related NCDs.“*

***Point 7.*** Improve - At the end of the Discussion, under the subheading "Limitations," review the limitations of your study.

***Response Ad 7.***

Thank you for your comment. We have reformulated and corrected this.

***Point 8****.* At the end of the limitations, under the subheading " Future directions".

***Response Ad 8.***

Thank you for your comment. We have added this.

**Point 9. Conclusion**

Take special care to draw your conclusions only from your results and verify that your conclusions are firmly supported by your data.

**Response Ad 9.**

Thank you for this comment. We have corrected this.

**Reviewers' comments**

**Reviewer's Responses to Questions**

**Comments to the Author**

1. If the authors have adequately addressed your comments raised in a previous round of review and you feel that this manuscript is now acceptable for publication, you may indicate that here to bypass the “Comments to the Author” section, enter your conflict of interest statement in the “Confidential to Editor” section, and submit your "Accept" recommendation.

Reviewer #1: All comments have been addressed

**Response:** Thank you.

Reviewer #2: All comments have been addressed

**Response:** Thank you.

2. Is the manuscript technically sound, and do the data support the conclusions?

The manuscript must describe a technically sound piece of scientific research with data that supports the conclusions. Experiments must have been conducted rigorously, with appropriate controls, replication, and sample sizes. The conclusions must be drawn appropriately based on the data presented.

Reviewer #1: Yes

**Response:** Thank you.

Reviewer #2: Yes

**Response:** Thank you.

3. Has the statistical analysis been performed appropriately and rigorously?

Reviewer #1: Yes

**Response:** Thank you.

Reviewer #2: Yes

**Response:** Thank you.

4. Have the authors made all data underlying the findings in their manuscript fully available?

The [PLOS Data policy](http://track.editorialmanager.com/CL0/http:%2F%2Fwww.plosone.org%2Fstatic%2Fpolicies.action%23sharing/1/010f0194f55db88b-b9e69bfb-1c8a-4e89-a9e4-e92ea0c29108-000000/YpSUAGNpau5f2cIJazxGfERu5oLYQYpa9Cl_lnbvQgA=197) requires authors to make all data underlying the findings described in their manuscript fully available without restriction, with rare exception (please refer to the Data Availability Statement in the manuscript PDF file). The data should be provided as part of the manuscript or its supporting information, or deposited to a public repository. For example, in addition to summary statistics, the data points behind means, medians and variance measures should be available. If there are restrictions on publicly sharing data—e.g. participant privacy or use of data from a third party—those must be specified.

Reviewer #1: Yes

**Response:** Thank you.

Reviewer #2: Yes

**Response:** Thank you.

5. Is the manuscript presented in an intelligible fashion and written in standard English?

PLOS ONE does not copyedit accepted manuscripts, so the language in submitted articles must be clear, correct, and unambiguous. Any typographical or grammatical errors should be corrected at revision, so please note any specific errors here.

Reviewer #1: Yes

**Response:** Thank you.

Reviewer #2: Yes

**Response:** Thank you.

**6. Review Comments to the Author**

Please use the space provided to explain your answers to the questions above. You may also include additional comments for the author, including concerns about dual publication, research ethics, or publication ethics. (Please upload your review as an attachment if it exceeds 20,000 characters)

**Reviewer #1**: Comments fully addressed thanks. I have no further comments to make. Congratulations on an excellent paper.

**Response:** Thank you.

**Reviewer #2:**

**Point 1.** Redundancy in Certain Statements:

Example: “Participants with BMI ≥ 25 had an increased risk of obesity.”

This is tautological, as BMI ≥ 30 already defines obesity.

Suggested Revision: “Participants with a BMI ≥ 25 were more likely to be overweight or obese.”

**Response** **Ad1:** Thank you for this comment. We have corrected this.

**Point 2.** Some Long or Complex Sentences Could Be Simplified for Readability:

**Response Ad2:** Thank you for this comment. We have shortened long sentences to improve readability.

**Point 3.** A few sentences contain multiple clauses, making them harder to follow.

Example:

Original: “Maintaining health requires strong motivation combined with positive lifestyle patterns such as eating healthy foods, exercising regularly, controlling stress, and abstaining from smoking and alcohol consumption.”

Suggested Revision: “Maintaining health requires motivation and positive lifestyle choices, including a balanced diet, regular exercise, stress management, and avoiding smoking and alcohol.”

**Response Ad3:** Thank you. We have corrected this according to your suggestion.

7. PLOS authors have the option to publish the peer review history of their article ([what does this mean?](https://track.editorialmanager.com/CL0/https:%2F%2Fjournals.plos.org%2Fplosone%2Fs%2Feditorial-and-peer-review-process%23loc-peer-review-history/1/010f0194f55db88b-b9e69bfb-1c8a-4e89-a9e4-e92ea0c29108-000000/NEkSI0JqkmAfnKArat3aKnK0PQz7QP6kFKRwm5IQi7s=197)). If published, this will include your full peer review and any attached files.

If you choose “no”, your identity will remain anonymous but your review may still be made public.

**Do you want your identity to be public for this peer review?** For information about this choice, including consent withdrawal, please see our [Privacy Policy](https://track.editorialmanager.com/CL0/https:%2F%2Fwww.plos.org%2Fprivacy-policy/1/010f0194f55db88b-b9e69bfb-1c8a-4e89-a9e4-e92ea0c29108-000000/3laUo16qDE0f6zAKamky4oOfDFOz_NuGTZb_6GK38jA=197).

Reviewer #1: **Yes:** Francis Finucane

Reviewer #2: **Yes:** Anees Alyafei

[NOTE: If reviewer comments were submitted as an attachment file, they will be attached to this email and accessible via the submission site. Please log into your account, locate the manuscript record, and check for the action link "View Attachments". If this link does not appear, there are no attachment files.]

While revising your submission, please upload your figure files to the Preflight Analysis and Conversion Engine (PACE) digital diagnostic tool, [https://pacev2.apexcovantage.com/](https://track.editorialmanager.com/CL0/https:%2F%2Fpacev2.apexcovantage.com%2F/1/010f0194f55db88b-b9e69bfb-1c8a-4e89-a9e4-e92ea0c29108-000000/2KrJ8NOR206X6iqN-Aui4jl-5MwapMfhBl1UNh85Udw=197). PACE helps ensure that figures meet PLOS requirements. To use PACE, you must first register as a user. Registration is free. Then, login and navigate to the UPLOAD tab, where you will find detailed instructions on how to use the tool. If you encounter any issues or have any questions when using PACE, please email PLOS at [figures@plos.org](mailto:figures@plos.org). Please note that Supporting Information files do not need this step.
