# Supplementary material for: The interplay between body mass index, motivation for food consumption, and noncommunicable diseases in the European population: A cross-sectional study
Source: PLoS One. 2025 May 14;20(5):e0322454. doi: 10.1371/journal.pone.0322454 (PMC12077776; doi:10.1371/journal.pone.0322454)
Supplement: Attachment — Submitted filename: Response_R3.docx [file pone.0322454.s007.docx]

**PONE-D-24-44681R2 - [EMID:cc0bfa0781d4c25a]**

The interplay between body mass index, motivation for food consumption, and noncommunicable diseases in the European population: a cross-sectional study

**Point 0.** Please review your reference list to ensure that it is complete and correct. If you have cited papers that have been retracted, please include the rationale for doing so in the manuscript text, or remove these references and replace them with relevant current references. Any changes to the reference list should be mentioned in the rebuttal letter that accompanies your revised manuscript. If you need to cite a retracted article, indicate the article’s retracted status in the References list and also include a citation and full reference for the retraction notice.

**Response Ad 0.** Thank you for your comment.

We have reviewed our reference list to ensure it is complete and accurate. We have not cited any retracted papers. The changes we have made are as follows:

- **Reference 2:** "Organisation" has been changed to "Organization."
- **Reference 3:** "2020;17:1–10" has been changed to "2020, 17:8878."
- **Reference 5:** In the journal title, the abbreviation "Heal" has been changed to "Health."
- **Reference 6:** The article and page number were missing and have now been updated to "2020, 22: e17640."
- **Reference 7:** The DOI was incorrect; the unnecessary "/S1" has been removed.
- **Reference 8:** The article and page number were missing and have now been updated to "2020, 20:1165." The DOI was also incorrect as it contained a reference to "/TABLES/3."
- **Reference 10:** The journal abbreviation was changed from "Lancet Public Heal" to "Lancet Public Health."
- **Reference 12:** Instead of just "559," the range "559-66" has been added.
- **Reference 14:** The year was incorrect and has now been changed to 2025.
- **Reference 15:** "Sarić MM" has been changed to "Matek Sarić M"; similarly, "Barić IC" is now written as "Colić Barić I."
- **Reference 16:** The year was "2022"; the volume and article are now correctly written as "2022; 2022:4243868." Additionally, "de la" is now correctly capitalized in the author's name.
- **Reference 18:** "Ridder D de" has been changed to "de Ridder D." The duplicate DOI has been removed, and the journal name "Psychol Health" has been added.
- **Reference 21:** The DOI has been added: <https://www.researchgate.net/publication/321142030>.
- **Reference 22:** "Guiné Raquel PF" has been changed to "Guiné RPF." The journal abbreviation has been changed from "Zdr. Varst" to "Slov J Public Heal," and page numbers "4-9" have been added.
- **Reference 23:** "Guine" has been changed to "Guiné." The third author's name has been corrected to "Szűcs V," and the fifth and sixth authors' names have been corrected to "Ljubičić M, Černelić-Bizjak M." Additionally, "2020, Vol IX, Page 888" has been removed.
- **Reference 26:** Page numbers "944-53" have been added.
- **Reference 28:** The unnecessary "2023 131" has been removed; instead of "1-10," it now states "2346."
- **Reference 29:** "2024; 14: e083443" has been added.
- **Reference 31:** A space has been added between "Pilot" and "Study." Page numbers have been corrected to "131-9."
- **Reference 33:** This was a duplicate of Reference 24, so it has been removed, and the numbering in the list has been adjusted.
- **Reference 34 (now 33):** "/TABLES/2" has been removed.
- **Reference 34:** Instead of "1-9," it now states "129," and "/TABLES/3" has been removed.
- **Reference 35:** "215,10" has been corrected to "2015; 10: e0118105."
- **Reference 42:** "315" has been corrected to "315-33."
- **Reference 43:** The authors are now correctly listed as "You HW, Tan PL, Mat Ludin AF." The DOI has been added: <https://doi.org/10.31436/imjm.v19i2.1567>.
- **Reference 44:** "1-9" has been corrected to "1354," and "/TABLES/1" has been removed.
- **Reference 46:** "Cult Atlas" has been changed to "Cultural Atlas," as it was not an abbreviation.
- **Reference 47:** "2023; 23" has been changed to "2023:1514."
- **Reference 48:** "2022; 13" has been changed to "2022; 13:873835." The incorrect DOI has been removed and replaced with the correct one.
- **Reference 50:** The incorrect link has been replaced with the correct one: <https://www.cdc.gov/healthy-weight-growth/healthy-eating/?CDC_AAref_Val=https://www.cdc.gov/healthyweight/healthy_eating/index.html>. The article title has been changed to *Tips for Healthy Eating for Healthy Weight 2023.*
- **Reference 51:** Instead of "1-9," it now states "1990." The incorrect DOI and "/TABLES/5" have been removed.
- **Reference 52:** The volume was incorrectly listed as 31 but should be 43.
- **Reference 53:** "2021; 19" has been changed to "2021; 19:320."
- **Reference 54:** "World Heal Organ" has been changed to "WHO."
- **Reference 56:** The reference has been revised for clarity:
  **Before:**
  *Ministry of Health of Portugal. “PNPAS” National Programme for the Promotion of Healthy Eating Portugal. Title in original language: Which “life stage(s)” for CVD prevention targets the intervention? What is the level of implementation of your example of good practice? n.d.* [*www.chrodis.eu*](http://www.chrodis.eu) *(accessed January 2, 2024).*
  **After:**
  *Ministry of Health of Portugal. “PNPAS” National Programme for the Promotion of Healthy Eating Portugal. n.d.* [*www.chrodis.eu*](http://www.chrodis.eu) *(accessed January 2, 2024).*
- **Reference 57:** "2020, 21" has been changed to "2020, 21; e13035."
- **References 60 and 61:** Removed, as this part of the conclusion was deleted in the revised article.

**Additional Editor Comments:**

(PLACE INSERTS IN A DIFFERENT COLOR FONT TO IDENTIFY CHANGES IN THE ARTICLE)

PUT IN RED LETTERS - ANSWER THE QUESTIONS BELOW POINT BY POINT.

INCLUDE THE PAGE AND LINE WHERE YOU ARE MAKING THE CHANGE.

PLEASE INCLUDE ALL REQUESTS IN THE MANUSCRIPT.

We followed the instructions and acted accordingly.

Thank you.

Include in article

**Point 1.** Rephrase the conclusion.

**Response Ad 1.** Thank you for your comment. We reformulated the conclusion**.**

**Point 2.** Include only what your data shows.

**Response Ad 2.** Thank you for your comment. We have ensured that conclusion include only findings directly supported by our data.

**Point 3.** The third paragraph of the conclusion should be in future directions as it is not a conclusion that your data shows.

**Response Ad 3.** Thank you for your feedback. We have revised the conclusion and moved the third paragraph to the "Future directions" section to ensure that the conclusion reflects only the findings supported by our data**.**
